# Molecular transmission network of pretreatment drug resistance among human immunodeficiency virus-positive individuals and the impact of virological failure on those who received antiretroviral therapy in China

**DOI:** 10.3389/fmed.2022.965836

**Published:** 2022-08-29

**Authors:** Hongli Chen, Jing Hu, Chang Song, Miaomiao Li, Yesheng Zhou, Aobo Dong, Ruihua Kang, Jingjing Hao, Jiaxin Zhang, Xiu Liu, Dan Li, Yi Feng, Lingjie Liao, Yuhua Ruan, Hui Xing, Yiming Shao

**Affiliations:** State Key Laboratory of Infectious Disease Prevention and Control (SKLID), National Center for AIDS/STD Control and Prevention (NCAIDS), Chinese Center for Disease Control and Prevention (China CDC), Collaborative Innovation Center for Diagnosis and Treatment of Infectious Diseases, Beijing, China

**Keywords:** HIV, pretreatment drug resistance, antiretroviral therapy, molecular transmission network, virological failure

## Abstract

**Objectives:**

We investigated the prevalence of pretreatment drug resistance (PDR), the molecular transmission network among HIV-positive individuals, and the impact of virological failure on those who received antiretroviral therapy (ART) in China.

**Methods:**

Based on the World Health Organization (WHO) surveillance guidelines for PDR, a baseline survey and follow-up were conducted in 2018 and 2021, respectively. Demographic information and plasma samples were obtained from all participants. HIV pol gene region sequences were used to analyze the PDR and molecular transmission networks using the Stanford HIV database algorithm and HIV-TRACE, respectively. This study assessed the odds ratios (OR) of PDR to virological failure (viral load ≥ 50 copies/mL) after 3 years of ART using multivariable logistic regression.

**Results:**

Of the 4,084 individuals, 370 (9.1%) had PDR. The prevalence of PDR to non-nucleoside reverse transcriptase inhibitors (5.2%) was notably higher than that to nucleoside reverse transcriptase inhibitors (0.7%, *p* < 0.001), protease inhibitors (3.0%, *p* < 0.001), and multidrug resistance (0.3%, *p* < 0.001). A total of 1,339 (32.8%) individuals from 361 clusters were enrolled in the molecular transmission network. Of the 361 clusters, 22 included two or more individuals with PDR. The prevalence of virological failure among HIV-positive individuals after 3 years of ART without PDR, those with PDR to Chinese listed drugs, and those with PDR to other drugs was 7.9, 14.3, and 12.6%, respectively. Compared with that in HIV-positive individuals without PDR, virological failure after 3 years of ART was significantly higher (OR: 2.02, 95% confidence interval (CI): 1.25–3.27) and not significantly different (OR: 1.72, 95% CI: 0.87–3.43) in individuals with PDR to Chinese listed drugs and those with PDR to other drugs, respectively. Missed doses in the past month were significantly associated with virological failure (OR, 2.82; 95% CI: 4.08–5.89).

**Conclusion:**

The overall prevalence of PDR was close to a high level and had an impact on virological failure after 3 years of ART. Moreover, HIV drug-resistant strains were transmitted in the molecular transmission network. These results illustrate the importance of monitoring PDR and ensuring virological suppression through drug adherence.

## Introduction

In 2008, the provision of free antiretroviral therapy (ART) for HIV/AIDs in China required the patient’s CD4 cell counts to be less than 200 cells/mm^3^; this was updated in 2014 to include patients with counts less than 500 cells/mm^3^. Since 2016, all people living with HIV can be treated with consent regardless of their CD4 cell counts and viral load level (1). As of 2021, there were 1,148,198 people living with HIV/AIDS in China, and 92.6% of those were receiving ART (2). With the rapid roll-out of ART, the case fatality rate of HIV infection has been effectively reduced, prolonging the life expectancy of HIV-positive individuals (3, 4). Unfortunately, patients receiving ART may develop drug resistance, which can be transmitted to others. The spread of drug-resistant strains is a persistent challenge to treating patients newly infected with HIV (5, 6).

Molecular transmission network technology is used to identify the maximum number of clusters and links based on genetic distance threshold using *pol* gene sequences (7–9). The results can guide the development of prevention and control measures. Using the tool to construct a molecular network in Southwest China identified rapidly growing drug resistance-related clusters containing the E138Q and V179D mutations (10). However, it is rarely used to explore the propagation of active clusters and the mutations present in the strains of HIV-positive individuals with pretreatment drug resistance (PDR) in China.

PDR refers to resistance detected among antiretroviral drug-naive patients initiating ART or those with previous antiretroviral drug exposure initiating or reinitiating first-line ART; PDR may be referred to as transmitted HIV drug resistance, acquired HIV drug resistance, or both (11). According to the World Health Organization (WHO), a drug resistance rate of < 5% is considered a low epidemic, ∼ 5% moderate epidemic, and > 15% a high epidemic (11). According to a recent survey, the PDR in HIV-infected patients in China ranges between 5 and 15% (7, 8, 12, 13). However, there has not been a large-scale investigation into the prevalence of HIV PDR in China. HIV virological suppression (defined as viral load < 50 copies/mL) is a key indicator of treatment success and is the most effective method for confirming the failure of ART regimens (14). Studies in Europe and sub-Saharan Africa have found that PDR impedes ART (15, 16). The effect of PDR on virological failure in China has not been estimated but may interfere with the achievement of “The Joint United Nations Program on HIV/AIDS” to diagnose 95% of all HIV patients, provide ART for 95% of those diagnosed, and achieve viral suppression in 95% of treated individuals by 2030 (95-95-95) (17). The present study investigated the prevalence of PDR in China and its impact on HIV virological failure among HIV-positive individuals after 3 years of ART. It also describes the active cluster transmission in China.

## Materials and methods

### Study design and participants

This large multicenter prospective cohort study was designed according to the WHO surveillance guidelines for the pretreatment of HIV drug resistance and pilot investigation in some regions of China in 2017 (8, 18). A total of 4,048 HIV-positive individuals in the baseline HIV PDR participated; the study was conducted in 30 provinces (municipalities or autonomous regions) of China in 2018, with a follow-up study conducted in 2021. Based on the manual of the National Free Antiretroviral Treatment, fourth edition (2016), ART adherence education and care was implemented during the study. Eligibility criteria included HIV-positive individuals aged ≥ 18 years who started ART in 2018. Patients were excluded if their plasma failed amplification sequences. All participants provided informed consent before enrollment.

### Data collection

Data were collected using a case-report form. Each study participant was assigned a confidential and unique identifier number, which was used as the coded identifier for the form and serum specimens. Baseline variables were collected for all HIV-positive individuals, including sociodemographic (age, sex, ethnicity, education, occupation, route of infection, marital status, and missed doses in the past month) and clinical characteristics (CD4 cell counts before ART, HIV genotype, PDR, initial ART regimen, and virus load after 3 years of ART).

### Laboratory tests

All CD4 cell counts and viral load tests were performed at the local Center for Disease Control and Prevention (CDC). Plasma was isolated and sent on dry ice to the WHO-certified drug resistance testing laboratory at the National Center for AIDS/STD Control and Prevention (NCAIDS), China CDC. Drug resistance testing was conducted for all plasma samples before treatment and plasma samples with a viral load ≥ 50 copies/mL measured 3 years after ART initiation. HIV RNA was extracted from 200 μL plasma and following the manufacturer’s protocol by using the QIAamp viral RNA mini kit (Qiagen, Hilton, Germany). Each batch of samples were added to positive, negative and blank control. HIV pol gene fragments was amplified by in-house sequencing and it has to covering the full-length protease (amino acids 1–99) and the first 240 amino acids of reverse transcriptase codons. The amplified products were sequenced using Sanger sequencing.

### Subtype and drug resistance analysis

Sequencer 4.10.1 (GeneCodes Corporation, Ann Arbor, MI, United States) was used for sequence splicing, the secondary peak threshold was set to 20% to identify ambiguities. The sequence was aligned using BioEdit (version 7.0.9, Informer Technologies Inc.). A phylogenetic tree with the neighbor-joining method was constructed using MEGA (version 6.06), reference sequences from HIV Databases^1^ and the bootstrap was set to 1,000, the check value was 70% to identify the subtype, control for potential laboratory contamination and sample contamination. Other sequence quality controls were monitored using the WHO HIVDR QC tool.^2^ We defined PDR using the Stanford HIV Drug Resistance Database.^3^ Drug susceptibility was classified into four categories depending on mutation score and degree: susceptible (< 15), low—(15–29), intermediate (30–59), and high-level (≥ 60) resistance. Drug resistance was defined by a drug resistance score ≥ 15 or drug resistance grade ≥ 3 for one or more of the 20 HIV antiretroviral drugs in the three categories of non-nucleoside reverse transcriptase inhibitors (NNRTIs), nucleoside reverse transcriptase inhibitors (NRTIs), and protease inhibitors (PIs) currently listed in the HIV database of Stanford University (18).

### Molecular transmission network inference

Sequences were excluded when the sequences contained ≥ 5% ambiguities or the sequence length < 1,000 bp. Workstation 15 Player was used to construct the Cent OS 7 platform and configure the transmission cluster engine (HIV-Trace) runtime environment (9). With the optimal threshold of 1.0% genetic distance, the HIV pol gene (location: 2,253–3,312 nt) sequence was compared with the HXB2 reference sequence, and the genetic distances of paired genes were calculated using the Tamura-NEI 93 model to construct the molecular network of PDR transmission (19). Each molecule in the network was represented by a node and matched with epidemiological information. Cytoscape (version 3.6.1) was used to process and generate the molecular network.

### Statistical analysis

Questionnaire data were double-entered and compared using EpiData software, version 3.1 (The EpiData Association, Odense, Denmark). Univariate and multivariate logistic regression models were used to estimate potential factors associated with virological failure among HIV-positive individuals after 3 years of ART. We adjusted for age, sex, ethnicity, education, occupation, marital status, route of infection, CD4 cell counts before ART, HIV genotype, and initial ART regimen. We constructed a multivariate logistic regression model stepwise to select variables independently associated with virological failure. All tests were two-tailed, and a *p*-value < 0.05 was considered statistically significant. Statistical analyses were performed using SAS V9.4 (SAS Institute, Inc., Cary, NC, United States).

### Ethical considerations

This study was approved by the ethics committee of the NCAIDS, China CDC. Written informed consent was obtained from all participants.

## Results

### Baseline characteristics of the study participants

Of the 4,084 individuals who completed the 2018 survey, 40.0% were aged 18–34 years, 82.9% were male, and 84.7% were of the Han ethnicity. Of these, 43.1% had received a senior high school education or above. Of the participants, 17.4% were farmers, 24.9% were unmarried; 50.2 and 40.8% were infected *via* heterosexual and homosexual intercourse, respectively, 2.5% were infected by intravenous drug use (IDU), and 6.5% were infected *via* other routes (6.5%). The percentage of individuals with CD4 cell counts before ART < 350 cells/mm^3^ was 60.9%. The primary genotype was CRF07_BC (40.8%). Additionally, 75% of the participants began ART with a regimen of tenofovir disoproxil fumarate (TDF) + lamivudine (3TC) + efavirenz (EFV)/nevirapine (NVP) (Table 1).

**TABLE 1:** Baseline characteristics of HIV-positive individuals in China, 2018.

Variable	*N*	%
Total	4,084	100.0
**Age (years)**		
18–34	1,635	40.0
35–49	1,289	31.6
≥50	1,160	28.4
**Sex**		
Male	3,386	82.9
Female	698	17.1
**Ethnicity**		
Han	3,460	84.7
Others	624	15.3
**Education**		
Illiteracy and primary school	1,274	31.2
Junior high school	983	24.1
Senior high school or above	1,762	43.1
Unknown	65	1.6
**Occupation**		
Farmer	709	17.4
Others	3,375	82.6
**Marital status**		
Unmarried	1,017	24.9
Married	1,307	32.0
Divorced/widowed	538	13.2
Missing	1,222	29.9
**Route of infection**		
Heterosexual intercourse	2,051	50.2
Homosexual intercourse	1,669	40.8
Intravenous drug use	100	2.5
Others	264	6.5
**CD4 cell counts before ART (cells/mm^3^)**		
<350	2,485	60.9
≥350	1,342	32.9
Missing	257	6.2
**HIV genotype**		
CRF01_AE	1,448	35.5
CRF07_BC	1,666	40.8
CRF08_BC	382	9.4
CRF55_01B	160	3.9
B	172	4.2
Others	256	6.2
**Initial ART regimen**		
AZT + 3TC + EFV/NVP	387	9.5
TDF + 3TC + EFV/NVP	3,063	75.0
AZT/D4T/TDF + 3TC + LPV/r	131	3.2
Others	503	12.3

### Pretreatment human immunodeficiency virus drug resistance and mutations

**FIGURE 1:**
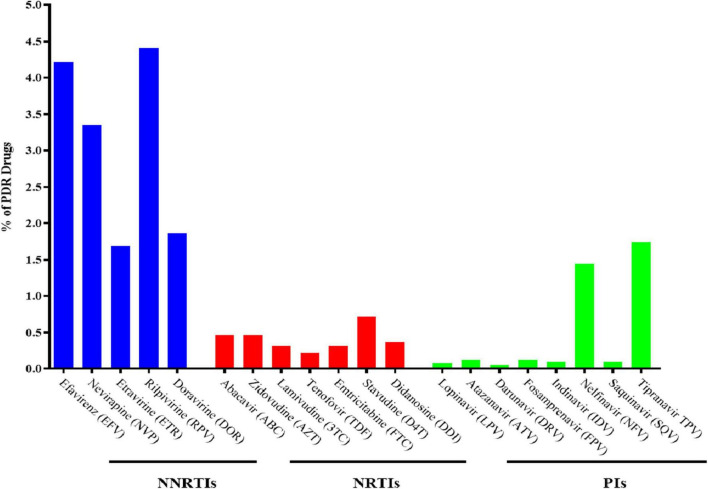
% of HIV PDR level to different antiretroviral drugs.

**FIGURE 2:**
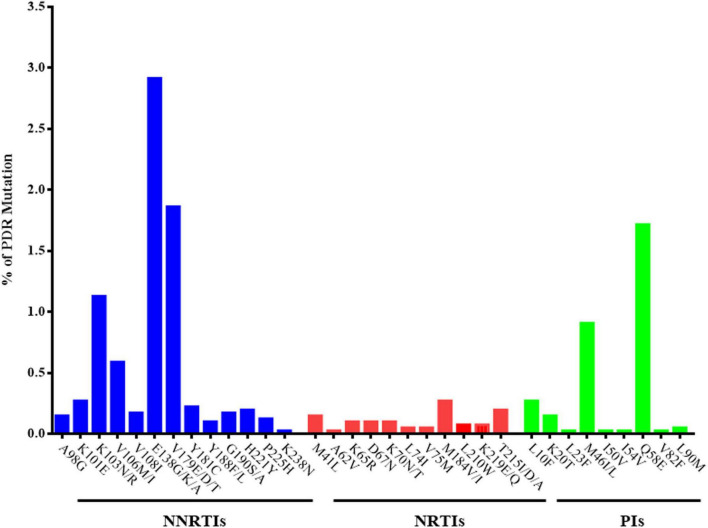
% of HIV PDR level to different drug resistance mutations.

The prevalence of PDR was 9.1% (370/4084, 95% CI 8.2–9.9%). The PDR to NNRTIs (205/4084, 5.2%) was notably higher than that to NRTIs (28/4084, 0.7%), PIs (124/4084, 3.0%), and multidrug resistance (13/4084, 0.3%). The prevalence of PDR with EFV, NVP, ETR, Rilpivirine (RPV), and Doravirine (DOR) was 4.2, 3.4, 1.7, 4.4, and 1.9%, respectively. Additionally, drug resistance mutations (DRMs), including E138G/K/A, V179E/D/T, K103N/R, V106M/I, and K101E, accounted for most of the NNRTI-related mutations. The most common PDR was that to (D4T) (29, 0.7%), followed by that to azidothymidine (AZT) (19, 0.5%) and abacavir (ABC) (19, 0.5%), whereas the most prevalent DRM was M184V (11, 0.3%) among NRTI-related mutations. Resistance to nelfinavir (NFV) (60, 1.5%) was the primary PDR within PIs agents, and the most prevalent PIs-related mutation was Q58E (70, 1.7%) (Figures 1, 2).

### Genetic transmission networks

**FIGURE 3:**
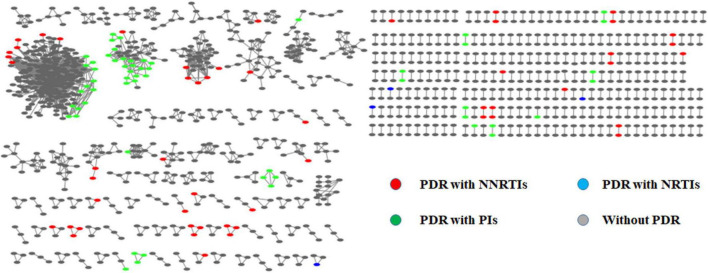
The molecular networks of pretreatment drug resistance.

Of the 4,084 individuals, 45 sequences were removed because the pol region was shorter than 1,000 bp. A total of 4,039 sequences were obtained for constructing a molecular transmission network. Under the optimal threshold of 1.0% genetic distance, 1,339 (32.8%) sequences (nodes) with a total of 361 clusters (size range: 2–299) were enrolled in the molecular transmission network. Among the 114 sequences with PDR, 46 clusters (22 clusters were from two or more PDR individuals), including the individuals with PDR to NNRTIs, NRTIs, and PIs, were 53, 4, and 57, respectively. The most frequently occurring DRM in the transmission network was Q58E (16.0%, 58/361). Eight molecular transmission networks of PDR (clusters of three or more PDR individuals with the same mutation) were distributed with the subtypes of CRF07_BC (4), CRF01_AE (2), CRF08_BC (1), and CRF55_01B (1) (Figure 3).

### Impact of pretreatment drug resistance on virological failure among HIV-positive individuals after 3 years of antiretroviral therapy

**FIGURE 4:**
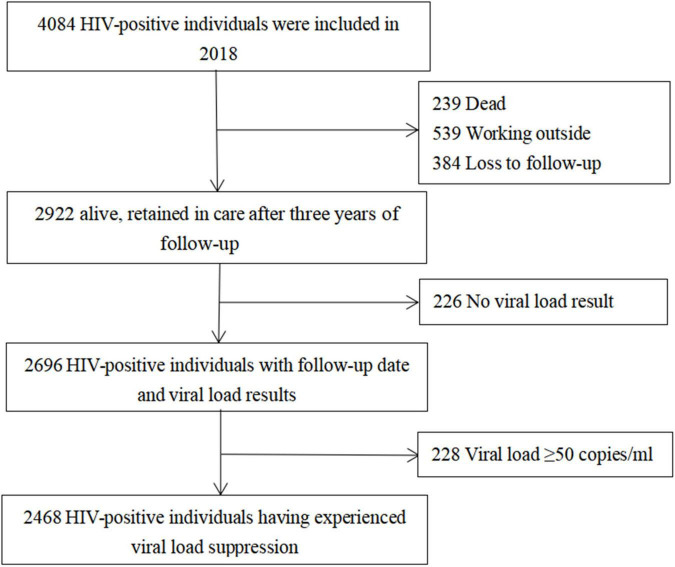
Flow chart.

Figure 4 summarizes the data for individuals selected in the cohort. Of the 4,084 individuals, the 3-year follow-up rate was 66.0% (2696/4084). Of 2,696 individuals in the cohort study, the median of follow-up person-years (IQR) was 3.07 (2.95–3.20). The prevalence of virological failure among HIV-positive individuals without PDR, those with PDR to Chinese listed drugs, and those with PDR to other drugs was 7.9, 14.3, and 12.6% after 3 years of ART, respectively. Compared with that in HIV-positive individuals without PDR, virological failure after 3 years of ART was significantly higher (OR: 2.02, 95% CI: 1.25–3.27) and no significant difference (OR: 1.72, 95% CI: 0.87–3.43) in individuals with PDR to Chinese listed drugs and those with PDR to other drugs, respectively. Missed doses in the past month were significantly associated with virological failure (OR: 2.82; 95% CI: 4.08–5.89) (Table 2).

**TABLE 2:** Impact of PDR on the virological failure among HIV-positive individuals after 3 years of ART.

Variable	*N*	Viral load ≥ 50 copies/mL	OR (*95*% CI)	*P*	AOR (95% CI)	*P*
		
		*N* (%)				
Total	2,696	228 (8.5)				
**PDR**						
Without PDR	2,441	193 (7.9)	1.00		1.00	
PDR to Chinese listed drugs^+^	168	24 (14.3)	1.91 (1.23–3.06)	0.004	2.02 (1.25–3.27)	0.004
PDR to other drugs*	87	11 (12.6)	1.69 (0.88–23.23)	0.115	1.72 (0.87–3.43)	0.122
**Missed doses in the past month**						
No	2,440	147 (6.8)	1.00		1.00	
Yes	256	81 (31.6)	4.28 (3.03–6.03)	0.000	4.08 (2.82–5.89)	0.000

Covariates of the adjusted model included: age, sex, ethnicity, education, occupation, marital status, route of infection, CD4 cell counts before ART, HIV genotype and initial ART regimen.

+: NNRTIs: EFV, NVP, ETR, RPV; NRTIs: ABC, AZT, 3TC, TDF, FTC, D4T; PIs: LPV/r, ATV/r, DRV/r.

*: NNRTIs: DOR; NRTIs: DDI; PIs: FPV/r, IDV/r, NFV, SQV/r, TPV/r.

## Discussion

This study found that 9.1% of Chinese HIV-positive individuals had PDR in 2018, which is a relatively high level according to the WHO. However, the overall PDR prevalence among HIV-positive individuals in China was 3.8% in 2004–2005, 4.7% in 2003–2014, 3.6% in 2015, and 6.8% in 2017, rapidly increasing over time (8, 20–22). The prevalence of PDR in China is lower than that in Japan (12.5%), Namibia (12.7%), Washington (22.5%), and Cameroon (10.4%) (23–26). Our study found that the prevalence of PDR to NNRTI was 5.0%. In China, the standard ART regimen consists of two NRTIs and one NNRTI. However, this protocol will need to be reviewed if the proportion of HIV-positive individuals with NNRTI resistance exceeds 10% (14). Our study outcomes were below the 10% threshold required for reviewing the recommended first-line ART regimens. Nonetheless, according to the 2019 WHO Global Drug Resistance Surveillance Report, the PDR of NNRTIs in 12 of the 18 monitored regions exceeded 10% (11). Interestingly, the prevalence of PDR to PIs was as high as 3.0% in our study, suggesting that the prevalence of PDR is related to tipranavir/ritonavir (TPV/r) being mainly used as a second-line regimen in China.

In our study, the most common DRMs found in the reverse transcription region were E138G/K/A, V179E/D/T, K103N/R, V106M/I, and K101E for NNRTIs; M184V/I and T215I/D/A for NRTIs; Q58E, and M46I/L for PIs. The E138G/K/A mutation (non-polymorphic) was not associated with susceptibility to resistance to any NNRTIs (27). The V179D mutation can cause drug resistance to several NNRTIs, and K103N (non-polymorphic) can induce resistance to NVP and EFV (28, 29). M184V was one of the most frequently detected PDR mutations, consistent with studies in sub-Saharan Africa and Ethiopia (30, 31). Mutations in Q58E (70/4084, 1.7%) and M46IL (37/4084, 0.9%) can cause drug resistance to TPV and NFV, respectively, and are recommended as second-line regimens in China. Of note, the current first-line regimen in China is TDF/AZT + 3TC + EFV/NVP (8). Conducting routine surveillance surveys for PDR and the development of resistant strains is necessary to guide the first-line regimens.

Our study constructed transmission networks to explore the presence of drug residence-related clusters in HIV-positive individuals before ART. Although the percentage of HIV-positive individuals with and without PDR belonging to the cluster did not significantly differ in transmission networks, among the 114 sequences with PDR found in 46 clusters, 22 clusters contained the same DRMs, and the two largest clusters were found in CRF07_BC. Among CRF07_BC strains with PDR, 52.8% (59/114) were within the network and distributed in ten clusters, indicating that the risk for CRF07_BC cluster growth should be monitored. Similar results were observed in some epidemic areas of China, indicating that drug-resistant HIV strains are transmitted among HIV-positive individuals (10, 32).

Our study also found that the overall prevalence of virological failure among HIV-positive individuals was 8.5% after 3 years of ART, notably lower than that in developing countries, such as Thailand (11.2%) and Uganda (14.3%) (33, 34). In addition, some countries disclosed by the WHO also fall short of the 95% target rate of virological suppression proposed by UNAIDS (11, 17). Several international studies have reported the effect of PDR on virological failure after ART (15, 16). Our study is the first to show that virological failure after 3 years of ART was higher in China, particularly in individuals with PDR to Chinese listed drugs. Moreover, PDR to Chinese listed drugs significantly impacted the virological failure of HIV-positive individuals after 3 years of ART. This could result from the wide-scale implementation of drug regimens to such a large population in China. In the future, this could lead to the emergence of drug-resistant HIV against some first-line drugs in HIV-positive individuals; further investigation is warranted. Missed doses in the past month were significantly associated with virological failure in our study. Adherence to ART is an important predictor of virological failure (33); previous studies have reported that poor adherence to ART can lead to rapid replication of drug-resistant HIV mutant strains (35, 36). Further studies on the factors influencing adherence to improve patient compliance are of national interest to the well-being of the health system (37). However, another survey found that the high prevalence of PDR was not associated with virological failure after ART, probably because patients were administered integrase strand transfer inhibitor (InSTI) regimens (one InSTI and two NRTIs) and had low levels of InSTI-related DRMs (29). We plan to conduct relevant studies on drug resistance of InSTI before ART in China in 2022.

This study resulted from large multicenter collaborations in 2018 and 2021; however, it had several limitations. First, Sanger sequencing cannot detect minority-resistant viral strains below 20% (38, 39), which could underestimate PDR prevalence. A recent study on infected Chinese patients in man who have sex with other men reported an ultra-deep sequencing-based method for DNA analysis that can help explore minority variants which, even at frequencies of 1%, can potentially affect virological responses (40). As a new technology, ultra-deep sequencing-based methods could help monitor the mutations and precisely capture population dynamics of HIV in China. Second, missed doses in the past month were based on self-reported measures, which could have been affected by recall bias. Third, only 66.0% of the 2018 cohort was followed up in 2021. The sample size was relatively small and lacked statistical power to analyze the effect of PDR on death. Therefore, the effect of PDR on virological failure may have been overestimated or underestimated. Fourth, due to the large number of sequences generated in routine HIV drug resistance testing and the small evolutionary variation of the pol gene sequence, China and the United States molecular network guidelines use the pol gene sequence to establish molecular networks (41, 42). However, in the case of multidrug-resistant viruses, this will affect the correctness of the molecular network. PDR in China is low, and its influence on the established molecular network may be relatively small. Finally, the env gene sequence can provide more information, but the amplification success rate is relatively low. At present, the number of env gene sequences is relatively small, and the env gene sequence has larger evolutionary variation, insertions, and deletions than the pol gene sequence. In the future, we will create a molecular network of the env gene sequence and compare it with that of the pol gene sequence.

## Conclusion

Our research illustrates that the prevalence of PDR in China is sufficient to call for a review of the standard ART protocol to manage the future burden of virological failure. Moreover, drug-resistant HIV strains were found to be transmitted in the molecular transmission network. Given the complexity and diversity of HIV/AIDS prevention and control in various regions of China, it is necessary to extend genotypic drug resistance testing to assess the level of drug resistance and explore ways to improve patient adherence to treatment protocols, ensuring optimal virological suppression of first-line regimens.

## Data availability statement

The original contributions presented in this study are included in the article/supplementary material, further inquiries can be directed to the corresponding author.

## Ethics statement

The studies involving human participants were reviewed and approved by the National Center for AIDS/STD Control and Prevention, Chinese Center for Disease Control and Prevention, China. The patients/participants provided their written informed consent to participate in this study. Written informed consent was obtained from the individual(s) for the publication of any potentially identifiable images or data included in this article.

## Author contributions

YR, HX, LL, YF, and YS were responsible for study design and planning. HC, YZ, AD, XL, and RK were responsible for statistics and figures. HC, JH, CS, ML, JJH, JZ, and DL conducted the experiments and collected the data. HC drafted the manuscript. YR and HX guided the whole study and revised the article. All authors read and approved the final version of the manuscript.

## Footnotes

1
https://www.hiv.lanl.gov/content/index

2
https://recall.bccfe.ca/who_qc/

3
https://hivdb.stanford.edu/hivdb/by-sequences/
